# Supplementary material for: Effect of Antimicrobial Use in Conventional Versus Natural Cattle Feedlots on the Microbiome and Resistome
Source: Microorganisms. 2023 Dec 14;11(12):2982. doi: 10.3390/microorganisms11122982 (PMC10745953; doi:10.3390/microorganisms11122982)
Supplement: Supplementary file 1 [file microorganisms-11-02982-s001.zip › microorganisms-2732065-supplementary.pdf]

Supplementals

Table S1. Pen level Metadata of animals used to collect fecal composite samples. Sex listed without "Yearling" are calves.

Pen-level Fecal Composite Sample ID	Age/Sex
Nat-Win-B-25Oct16-M1	Yearling, Steers
Nat-Win-B-25Oct16-M2	Yearling, heifers
Con-Win-A-22Oct16-M4	Yearling, Steers
Nat-Win-C-31Jan17-M7	Steers
Con-Win-D-1Feb17-M9	Yearling, Steers
Con-Win-D-2Feb17-M10	Yearling, Heifers
Nat-Sum-B-28Jun17-M17	Yearling steers
Nat-Sum-B-28Jun17-M18	Yearling heifers
Nat-Win-B-1Mar17-MG2	Yearling, Heifers
Con-Win-A-1Mar17-MG3	Yearling, Not segregated
Con-Win-A-1Mar17-MG4	Yearling, Not segregated
Nat-Sum-B-26Apr17-MG5	Yearling, Steers
Nat-Sum-B-26Apr17-MG6	Yearling, Heifers
Con-Sum-A-26Apr17-MG7	Yearling, Heifers
Con-Sum-A-26Apr17-MG8	Yearling, Steers
Nat-Sum-C-10Apr18-MG9	Steers
Nat-Sum-C-10Apr18-MG10	Heifers
Con-Sum-D-10Apr18-MG11	Yearling, Not segregated
Con-Sum-D-10Apr18-MG12	Yearling, Not segregated
Nat-Win-B-13Mar18-MG13	Not segregated
Nat-Win-B-13Mar18-MG14	Not segregated
Con-Win-A-13Mar18-MG15	Yearling, Not segregated
Con-Win-A-13Mar18-MG16	Yearling, Not segregated
Nat-Sum-C-26Jun18-MG17	Steers
Nat-Sum-C-26Jun18-MG18	Heifers
Con-Sum-D-26Jun18-MG19	Yearling, Not segregated
Con-Sum-D-26Jun18-MG20	Yearling, Not segregated
Nat-Sum-B-25Jul18-MG21	Yearling, Steers
Nat-Sum-B-25Jul18-MG22	Yearling, Heifers
Con-Sum-A-25Jul18-MG23	Yearling, Not segregated
Con-Sum-A-25Jul18-MG24	Yearling, Not segregated
Nat-Sum-C-1Aug17-MG25	Heifers
Nat-Sum-C-1Aug17-MG26	Heifers
Con-Sum-D-1Aug17-MG27	Yearling, Heifers
Con-Sum-D-1Aug17-MG28	Yearling, Steers
Nat-Win-C-24Oct17-MG29	Heifers
Con-Win-D-24Oct17-MG31	Yearling, Steers
Con-Win-D-24Oct17-MG32	Yearling, Heifers
Nat-Win-C-29Jan18-MG33	Heifers
Nat-Win-C-29Jan18-MG34	Heifers
Con-Win-D-29Jan18-MG35	Yearling, Heifers
Con-Win-D-29Jan18-MG36	Yearling, Steers

Nat-Sum-B-05Sep17-MG41	Yearling, Steers
Nat-Sum-B-05Sep17-MG42	Yearling, Heifers
Con-Sum-A-05Sep17-MG43	Yearling, Steers
Con-Sum-A-05Sep17-MG44	Yearling, Heifers
Nat-Sum-B-29May18-MG45	Yearling, no data
Nat-Sum-B-29May18-MG46	Yearling, no data
Con-Sum-A-29May18-MG47	Yearling, Heifers
Con-Sum-A-29May18-MG48	Yearling, Steers
Con-Win-A-25Oct16-MG49	Yearling, Heifers
Nat-Win-C-31Jan17-MG50	Heifers
Nat-Win-B-11Dec17-MG53	Yearling, Steers
Nat-Win-B-11Dec17-MG54	Yearling, Heifers
Con-Win-A-11Dec17-MG55	Yearling, Steers
Con-Win-A-11Dec17-MG56	Yearling, Heifers
Nat-Win-C-29Mar17-MG57	Heifers
Nat-Win-C-29Mar17-MG58	Steers
Con-Win-D-29Mar17-MG59	Yearling, Heifers
Con-Win-D-29Mar17-MG60	Yearling, Steers

Table S2. Most abundant archaeal and bacterial classes (>1%) and orders (top 20) of fecal and catch basin water samples with mean normalized abundance, interpreted significance of adjusted p-value (q>0.05 = ns; q<0.05 = *; q<0.01 = **; q<0.001 = ***; q<0.0001 = ****), and fold change for significant differences from natural (NAT) to conventional (CONV).

Sample Type	Taxa Level	Taxon	CONV Mean Normalized Abundance	NAT Mean Normalized Abundance	Interpreted Significance	Fold Change
Fecal Composite	Class	Actinobacteria	0.055	0.066	**	1.09
		Alphaproteobacteria	0.041	0.045	ns	-
		Bacilli	0.078	0.091	*	1.06
		Bacteroidia	0.210	0.168	***	0.81
		Betaproteobacteria	0.031	0.033	ns	-
		Chlorobia	0.002	0.002	ns	-
		Clostridia	0.271	0.307	ns	-
		Coriobacteriia	0.012	0.014	ns	-
		Cytophagia	0.014	0.014	ns	-
		Deltaproteobacteria	0.016	0.017	ns	-
		Epsilonproteobacteria	0.012	0.009	****	0.97
		Flavobacteriia	0.039	0.038	*	0.93
		Gammaproteobacteria	0.096	0.091	*	0.91
		Methanobacteria	0.014	0.006	***	0.58
		Mollicutes	0.007	0.009	**	1.11
		Negativicutes	0.014	0.012	ns	-
		Spirochaetia	0.029	0.015	**	0.69
	Order	Alteromonadales	0.009	0.008	*	0.88
		Bacillales	0.051	0.059	*	1.05
		Bacteroidales	0.210	0.167	***	0.80
		Bifidobacteriales	0.006	0.011	***	1.33
		Burkholderiales	0.023	0.024	ns	-

		Campylobacterales	0.012	0.009	****	0.77
		Caulobacterales	-	-	-	-
		Chromatiales	-	-	-	-
		Clostridiales	0.266	0.301	ns	-
		Corynebacteriales	0.012	0.013	ns	-
		Cytophagales	0.014	0.014	ns	-
		Desulfovibrionales	-	-	-	-
		Enterobacterales	0.026	0.024	*	0.90
		Erysipelotrichales	0.010	0.011	ns	-
		Flavobacteriales	0.040	0.039	*	0.92
		Lactobacillales	0.026	0.030	*	1.04
		Methanobacteriales	0.014	0.006	***	0.57
		Micrococcales	0.009	0.010	ns	-
		Nitrosomonadales	-	-	-	-
		Pseudomonadales	0.020	0.020	ns	-
		Rhizobiales	0.016	0.018	ns	-
		Rhodobacterales	-	-	-	-
		Rhodocyclales	-	-	-	-
		Sphingomonadales	-	-	-	-
		Spirochaetales	0.027	0.013	**	0.66
		Streptomycetales	0.009	0.010	ns	-
		Xanthomonadales	-	-	-	-
Catch Basin Water	Class	Actinobacteria	0.096	0.122	*	0.99
		Alphaproteobacteria	0.154	0.116	ns	-
		Bacilli	0.017	0.029	****	1.06
		Bacteroidia	0.019	0.041	****	1.01
		Betaproteobacteria	0.229	0.251	ns	-
		Clostridia	0.018	0.057	ns	-
		Cytophagia	0.009	0.015	ns	-
		Deltaproteobacteria	0.105	0.08	ns	-
		Epsilonproteobacteria	0.018	0.015	ns	-
		Flavobacteriia	0.030	0.041	****	1.13
		Gammaproteobacteria	0.249	0.161	ns	-
		Methanobacteria	0.000	0.002	****	1.18
	Order	Bacillales	0.012	0.02	ns	-
		Bacteroidales	0.018	0.04	ns	-
		Burkholderiales	0.174	0.202	ns	-
		Campylobacterales	0.019	0.015	ns	-
		Caulobacterales	0.017	0.01	ns	-
		Chromatiales	0.085	0.016	ns	-
		Clostridiales	0.017	0.055	ns	-
		Corynebacteriales	0.018	0.022	ns	-
		Desulfovibrionales	0.086	0.062	ns	-
		Enterobacterales	0.019	0.018	ns	-
		Flavobacteriales	0.031	0.042	ns	-
		Micrococcales	0.022	0.025	ns	-
		Nitrosomonadales	0.011	0.02	ns	-
		Pseudomonadales	0.062	0.056	ns	-
		Rhizobiales	0.052	0.045	ns	-

		Rhodobacterales	0.047	0.031	ns	-
		Rhodocyclales	0.038	0.023	ns	-
		Sphingomonadales	0.021	0.015	ns	-
		Streptomycetales	0.023	0.026	ns	-
		Xanthomonadales	0.018	0.016	ns	-

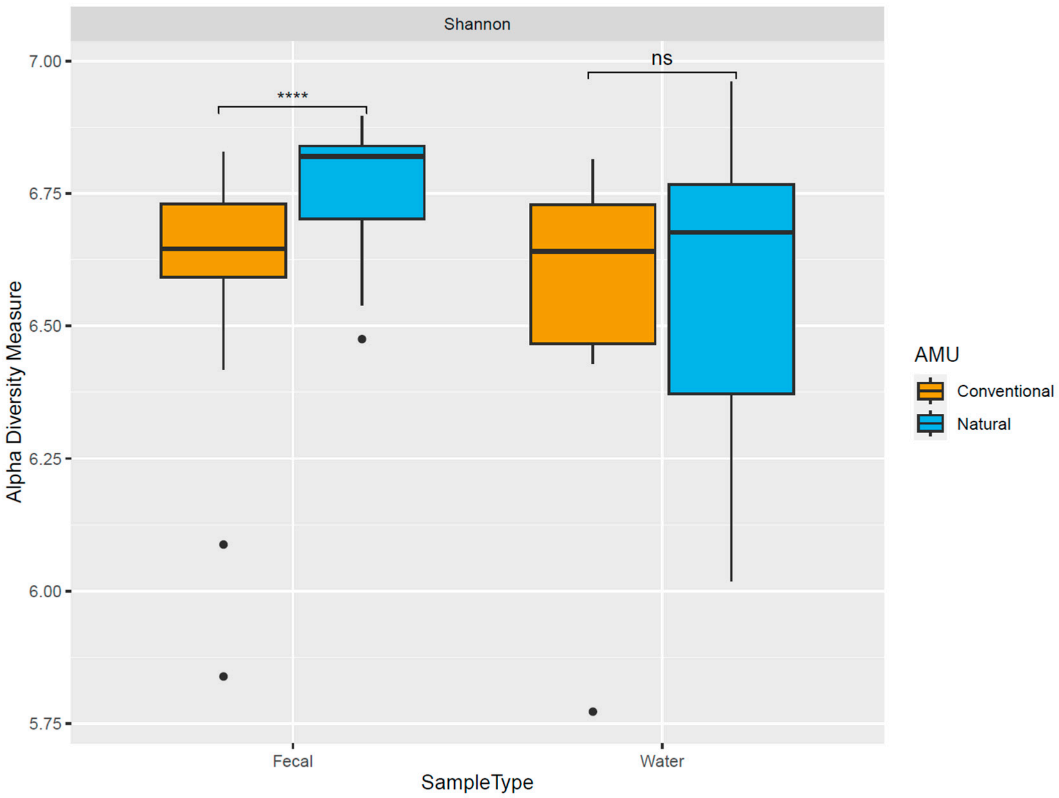

Figure S1. Boxplot of Shannon index comparisons between conventional and natural feedlots for fecal and catch basin water samples. (Wilcoxon signed rank test; p>0.05 =ns; p<0.0001 = ****)

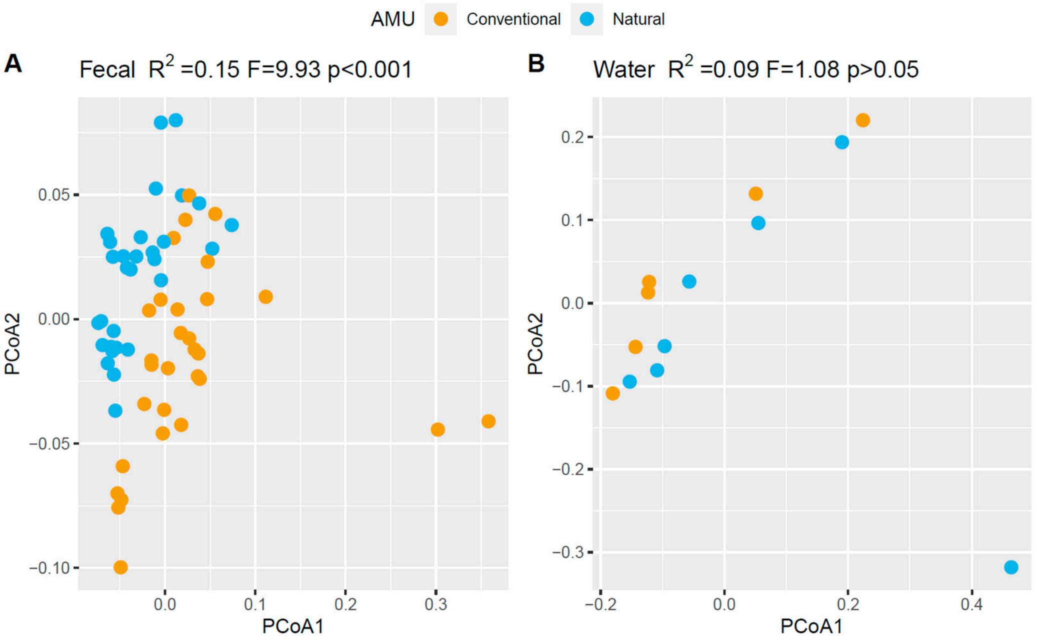

Figure S2. Principle component analyses (PCoA) of beta diversity differences using Bray-Curtis for fecal (**A**) and catch basin water (**B**) samples between conventional and natural feedlot types. (PERMANOVA test)

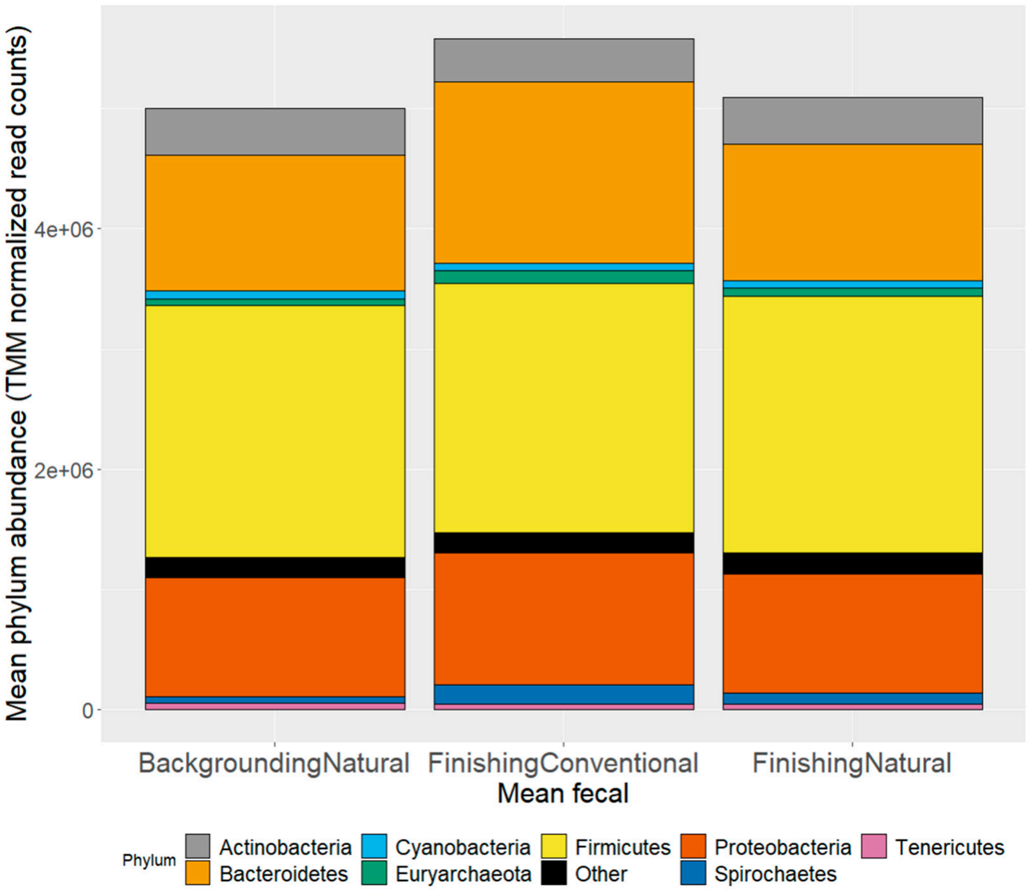

Figure S3. Stacked bar plots comparing TMM (trimmed mean of m-values) normalized mean read counts per sample of prevalent phyla (>1%) across fecal sample diet (Backgrounding vs Finishing) and feedlot management practices (Conventional vs Natural).

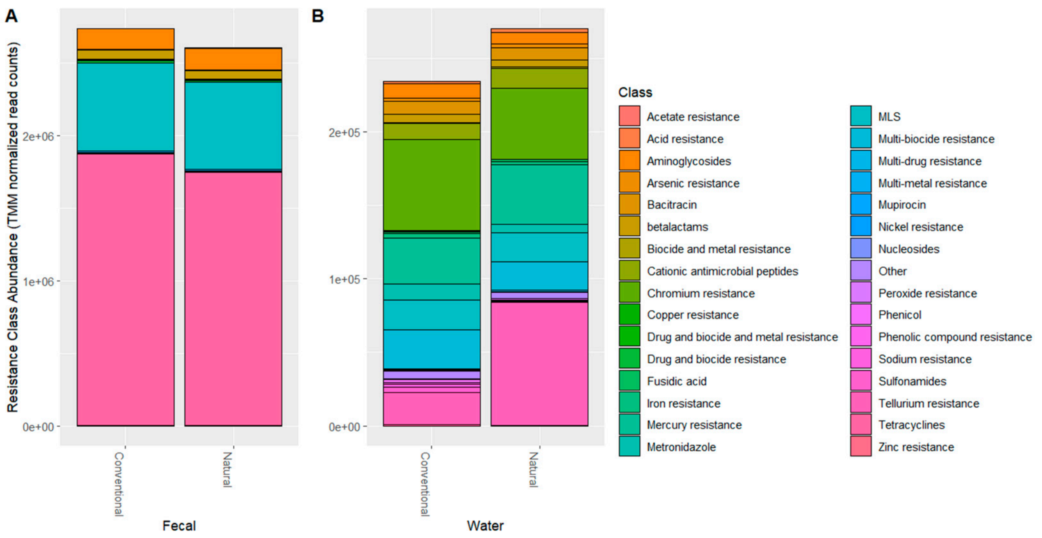

Figure S4. Stacked bar plots of TMM (trimmed mean of m-values) normalized read counts per feedlot management practice for fecal (A) and catch basin water (B) antimicrobial resistance gene classes.

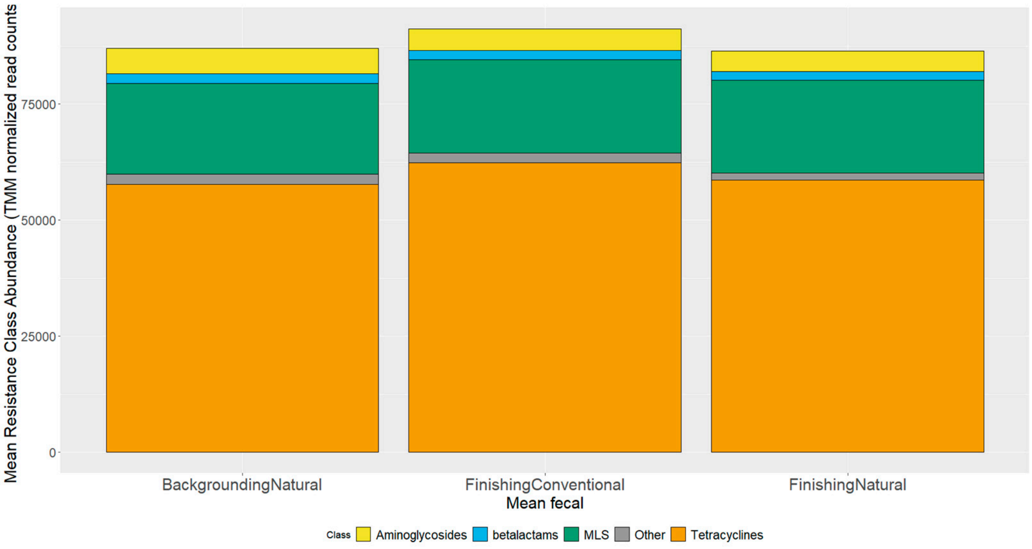

Figure S5. Stacked bar plots comparing TMM (trimmed mean of m-values) normalized mean read counts per sample of prevalent antimicrobial resistance gene classes (>1%) across fecal sample diet (Backgrounding vs Finishing) and feedlot management practices (Conventional vs Natural).
